# Sense and specificity in neuronal calcium signalling^☆^

**DOI:** 10.1016/j.bbamcr.2014.10.029

**Published:** 2015-09

**Authors:** Robert D. Burgoyne, Lee P. Haynes

**Affiliations:** Department of Cellular and Molecular Physiology, The Physiological Laboratory, Institute of Translational Medicine, University of Liverpool, Crown Street, Liverpool, L69 3BX, United Kingdom

**Keywords:** CaBP, calcium-binding protein, DREAM, downstream regulatory element antagonist modulator, CDF, Ca^2 +^-dependent facilitation, CDI, Ca^2 +^-dependent inhibition, GCAP, guanylyl cyclase activating protein, IL1RAPL1, interleukin 1 receptor accessory protein-like 1 protein 1, KChIP, potassium channel interacting protein, NCS, neuronal calcium sensor, NCX, sodium/calcium exchanger, VGCC, voltage-gated Ca^2 +^ channel, VILIP, visinin-like protein, *Caenorhabditis elegans*, Ca^2 +^-binding proteins, Ca^2 +^ channel, Ca^2 +^ sensors, NCS-1, Neuronal signalling

## Abstract

Changes in the intracellular free calcium concentration ([Ca^2 +^]_i_) in neurons regulate many and varied aspects of neuronal function over time scales from microseconds to days. The mystery is how a single signalling ion can lead to such diverse and specific changes in cell function. This is partly due to aspects of the Ca^2 +^ signal itself, including its magnitude, duration, localisation and persistent or oscillatory nature. The transduction of the Ca^2 +^ signal requires Ca^2 +^ binding to various Ca^2 +^ sensor proteins. The different properties of these sensors are important for differential signal processing and determine the physiological specificity of Ca^2 +^ signalling pathways. A major factor underlying the specific roles of particular Ca^2 +^ sensor proteins is the nature of their interaction with target proteins and how this mediates unique patterns of regulation. We review here recent progress from structural analyses and from functional analyses in model organisms that have begun to reveal the rules that underlie Ca^2 +^ sensor protein specificity for target interaction. We discuss three case studies exemplifying different aspects of Ca^2 +^ sensor/target interaction. This article is part of a special issue titled the 13th European Symposium on Calcium.

## Highlights

• Ca^2 +^ regulates multiple aspects of neuronal function over varying time scales.
• The specificity of Ca^2 +^ signalling is determined by the properties of Ca^2 +^ sensors
• Differing properties of closely related Ca^2 +^ sensors contributes to specificity.
• The structure of Ca^2 +^ sensors determines specificity of target protein interactions.
• We discuss three case studies involving calmodulin, NCS proteins and CaBP proteins.
• The examples illustrate the structural–function relationships of Ca^2 +^ sensors.

## 1 Introduction

The intracellular free calcium concentration ([Ca^2 +^]_i_) is tightly regulated through multiple mechanisms in neurons [1], and changes in [Ca^2 +^]_i_ have crucial roles in the control of normal neuronal function [2]. In addition, abnormalities in Ca^2 +^ signalling have been implicated in many aspects of neuropathology, neurodegeneration [3], [4], [5] and psychiatric disorders [6], [7]. The mechanisms that elevate [Ca^2 +^]_i_ include entry of extracellular Ca^2 +^ through voltage-gated Ca^2 +^ channels and release from intracellular stores such as the endoplasmic reticulum (ER), lysosomes and mitochondria. [Ca^2 +^]_i_ can be reduced by sequestration into the same cellular organelles and by extrusion across the plasma membrane and these mechanisms have been well characterised [1], [8]. Large numbers of studies on the localisation, magnitude and time course of [Ca^2 +^]_i_ fluxes in neurons have been published. Changes in [Ca^2 +^]_i_ can be local or global, highly transient, oscillatory or persistent. Localisation of [Ca^2 +^]_i_ changes to particular neuronal compartments is important for the generation of specific neuronal responses. For example, certain stimuli strictly require nuclear rather than cytoplasmic changes in [Ca^2 +^]_i_ [9] and also highly localised [Ca^2 +^]_i_ changes restricted to dendritic spines may play an important role in activity-dependent synaptic plasticity [10], [11]. The physiological effects of elevation of [Ca^2 +^]_i_ can be manifest in microseconds (neurotransmitter release), milliseconds (channel facilitation or inactivation) or over much longer time scales leading to changes in gene expression [12], [13] and effects on synaptic plasticity [14], neuronal development [15], learning [16], neuronal survival and cell death.

Clearly Ca^2 +^ can influence many aspects of neuronal function with the same fundamental signalling ion being used to produce a variety of subtle and distinct changes. It is known, for example, that Ca^2 +^ entry through different types of plasma membrane channels can affect changes in gene expression by different modes of signalling [17], [18]. Of note here is the fact that even different classes of voltage-gated Ca^2 +^ channels (VGCCs) in the same neurons are coupled through distinct signalling pathways to changes in gene expression [19]. Ca^2 +^ signals are translated into changes in cellular function through various types of Ca^2 +^ sensor proteins that in general terms detect increases in [Ca^2 +^]_i_ by becoming loaded with Ca^2 +^, undergo a conformational change and then interact with and regulate various target proteins. The subtlety of neuronal Ca^2 +^ signalling is underpinned by differential signal processing by these Ca^2 +^ sensor proteins, which in turn determines the physiological specificity of Ca^2 +^ signalling pathways.

Neurons express a large number of Ca^2 +^ sensor proteins [20] ranging from synaptotagmin I, which is the essential Ca^2 +^-sensor for fast (microsecond) neurotransmitter release [21], through the annexins [22], to many different EF hand containing proteins [20]. The EF hand is a highly conserved Ca^2 +^-binding motif [23] which is present, for example, in the ubiquitous Ca^2 +^ sensor protein calmodulin [24]. Calmodulin has numerous targets for regulation and is known to have multiple functions in neurons acting via various targets including Ca^2 +^/calmodulin-dependent protein kinase II [25]. Other EF hand containing Ca^2 +^ sensors expressed in neurons include the calcium-binding protein (CaBP) family [26], [27] and the neuronal calcium sensor (NCS) proteins [28], [29], [30].

The differential processing of neuronal Ca^2 +^-signals is affected by multiple aspects of the properties of Ca^2 +^ sensors, and we suggest that they have a key role in determining signalling specificity. Factors that influence differential Ca^2 +^ signalling include varied expression levels of sensors between neuronal cell types [31], differences in subcellular localisation, affinity and dynamics of Ca^2 +^-binding [32], association with protein signalling complexes [33], [34], regulation and variations in stoichiometry of binding with target proteins [35], [36] and specificity of target protein interaction. Appreciation of the contribution of Ca^2 +^-sensors to signalling specificity requires analysis of both the Ca^2 +^-sensors and their target interactions at a structural level [24], [37]. The rules underlying the sensing and specificity of Ca^2 +^ signalling in neurons have begun to emerge in recent years but still remain to be fully understood. In this review, we will present three case studies to illustrate current understanding of the molecular and structural basis of the contribution of the properties of Ca^2 +^ sensors to differential neuronal signal processing.

## 2 Case Study 1: NCS-1, a Ca^2 +^ sensor with multiple specific targets

NCS-1 is a member of the neuronal calcium sensor family that is encoded by 14 genes in mammals. Of these, recoverin and the guanylyl cyclase activating protein (GCAP) 1–3 have specialised roles in the Ca^2 +^ regulation of phototransduction. One other subfamily, consisting of the visinin-like protein (VILIP) 1–3, neurocalcin δ and hippocalcin has less well-defined functions. Hippocalcin, as suggested by its name, has a very restricted pattern of expression mainly in hippocampal neurons where it shows dynamic membrane association (the Ca^2 +^ myristoyl switch [38]) in response to Ca^2 +^-elevation [39] and neuronal activity [40], [41], including activity-dependent translocation into dendritic spines. Hippocalcin has been implicated as a Ca^2 +^ sensor in long-term depression [42], [43] and the gating of channels underlying a slow after-hyperpolarisation current [44]. VILIPs also show the Ca^2 +^ myristoyl switch [45] and may have multiple roles, including the regulation of receptor trafficking [46]. The potassium-channel interacting proteins (KChIPs) have all been implicated in the gating [47] and trafficking [48] of A-type potassium channels but show differential expression in different classes of neurons [49], [50]. One of them, KChIP3 also known as calsenilin/DREAM, regulates presenilin function [51] and can act as a transcriptional repressor [52]. The other KChIPs may share the DREAM activity [53], but KChIP3 is specific among the KChIPs in increasing regulated secretion and down regulating the Na^+^/Ca^2 +^ exchanger NCX3 [54].

NCS-1 appears to be expressed in most neuronal cell types [55], [31], and studies in various organisms have determined that it has multiple physiological functions [29], [56], [57], [58]. Some of the roles of NCS-1 may be specific to certain organisms such as its particular role in temperature-dependent behaviours in *Caenorhabditis elegans* as a consequence of its restricted neuronal expression [59], [60], [61]. One of two encoded NCS-1 proteins generated through gene duplication (ncs-1a) is required for semi-circular canal formation in the zebrafish inner ear [62] and NCS-1 (frequenin, Frq) is required for the development of synaptic boutons in *Drosophila* [56], the organism in which NCS-1 was first discovered [63]. In addition, in *Saccharomyces cerevisiae* NCS-1 (Frq1) is essential for survival as a consequence of its requirement for activation of the phosphatidylinositol-4-kinase Pik1 [64] despite its absence not being lethal in other organisms. In mammalian cells, NCS-1 regulates Ca^2 +^-dependent exocytosis [65], long-term depression [66], axonal growth and neuronal regeneration [67] and channel function [68]. In mice, knock-out of NCS-1 has relatively subtle effects but results in an increase in anxiety and depressive behaviour [69]. Selective overexpression of NCS-1 in adult mouse dentate gyrus neurons promoted a form of exploratory behaviour and enhanced acquisition of spatial memory [70].

**Fig. 1:**
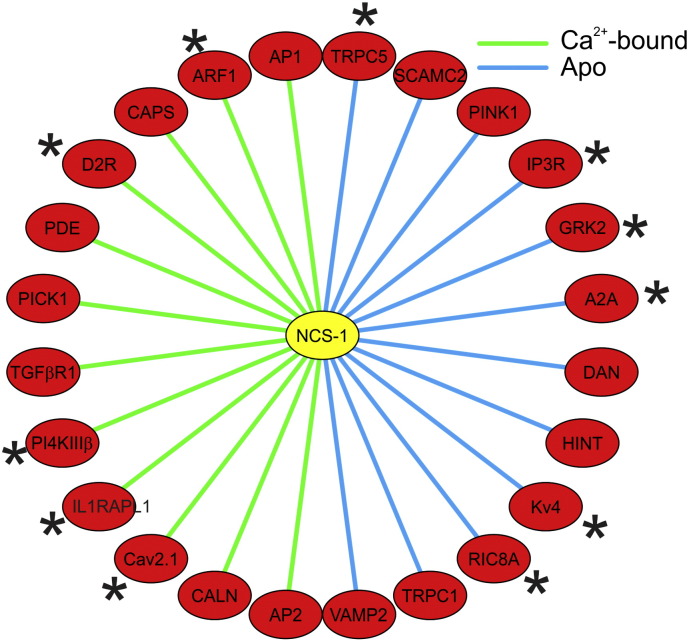
Known target proteins for NCS-1 indicating interactions that require the Ca^2 +^-bound or the apo form of NCS-1. The interactions shown include ones that are based only *in vitro* binding assays as well as interactions that have been substantiated and shown to have physiological relevance in functional studies (marked with an asterisk).

**Fig. 2:**
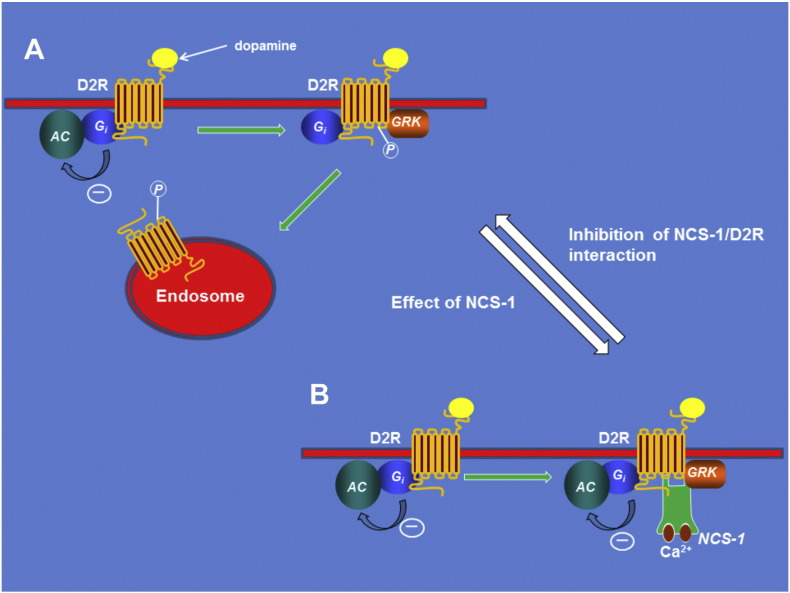
The role of NCS-1 in the regulation of dopamine D2 receptors. Agonist occupancy of the receptors results in inhibition of adenylate cyclase (AC) through the inhibitory G-protein Gi. In addition, dopamine D2 receptors undergo desensitisation following agonist binding due to their internalisation by endocytosis. This requires phosphorylation of the receptor by a G-protein coupled receptor kinase (GRK). The role of NCS-1 is to bind both the D2 receptor C-terminal tail and also GRK. Phosphorylation of the receptor is inhibited by NCS-1 and it is not internalised but instead remains at the cell surface. Inhibition of NCS-1/D2R interaction will allow receptor desensitisation.

NCS-1 has many known interacting partners [71] (Fig. 1), some of which are unique for NCS-1 but some that are also regulated by other Ca^2 +^ sensors particularly calmodulin [72]. Some of the interactions are known only from *in vitro* binding assays, and so their biological importance is unclear. It is possible that those binding partners that are also calmodulin targets *in vitro* [72] are not regulated by NCS-1 under physiological conditions [73]. There are, however, several physiological effects of NCS-1 that can be attributed directly to one of its identified target proteins (Table 1).Two documented NCS-1 interactions are of possible clinical significance. The potential importance of the regulation of dopamine D2 receptors by NCS-1 whereby NCS-1 inhibits D2 receptor internalisation (Fig. 2) [74] stems from the fact that dopamine is of key importance for signalling within the CNS and in addictive behaviour [75], [76]. The regulation of D2 receptors by NCS-1 has been shown to underlie the effect of overexpression of NCS-1 on spatial memory acquisition [70]. Importantly, dopamine D2 receptors are the targets for all known effective antipsychotic drugs [77]. Interestingly, NCS-1 is up-regulated in patients with bipolar disorder or schizophrenia [78] and in response to anti-psychotic drugs [79] and is genetically associated with cocaine addiction [80] believed to be linked to effects of cocaine on dopamine transporters [81]. Recently, NCS-1 has been shown to be required for an adaptive response to dopaminergic agonists in substantia nigra neurons, and coupled with its up-regulation in the substantia nigra from patients with Parkinson's disease, this has resulted in the suggestion that it could be a target for modifying the vulnerability of neurons in the substantia nigra to neurodegeneration [82]. The binding of NCS-1 to the D2 receptor involves the very short cytoplasmic C-terminal domain of the receptor [74]. This interaction has been partially characterised using structural approaches [83] and this may allow exploration of the interaction as a therapeutic drug target.

The other clinically important interaction is with the interleukin 1 receptor accessory protein-like 1 protein (IL1RAPL1), which appears to be specific for NCS-1 [84]. Mutations in ILIRAPL1 have been shown to result in X-linked mental retardation [85], [86] and also have been linked to autistic spectrum disorder (ASD, [87]). Interestingly, the latter study also identified a mutation (R120Q) within NCS-1 in an individual with ASD. This mutation was found to cause a functional deficit in NCS-1 [88] that appeared to be related to a change in the structural dynamics of the C-terminus of the protein [88]. However, the physiological relevance of this mutation and its exact relationship to the disease phenotype remain to be established.

One key question is, what determines the specificity of target recognition for NCS-1 over other Ca^2 +^ sensors and more particularly other members of the NCS protein family which have their own specific targets? The NCS proteins show differences in membrane-association with some such as recoverin and hippocalcin showing transient membrane-association through their Ca^2 +^/myristoyl switch mechanism [38], [39], [45], whereas others including KChIP3 and KChIP4 are predominantly cytoplasmic [54]. Others including KCIP1 and mammalian NCS-1 appear to be constitutively membrane targeted due to their N-terminal myristoylation [89], [90], but note, however, that Ncs-1 from fission years has been shown to have the Ca^2 +^/myristoyl switch [91]. The NCS proteins can, however, be very closely related to each other at a protein sequence level (> 60% identity). Structural analyses of several NCS proteins have shown common features with highly similar main chain topologies [37]. The gross tertiary topology of NCS proteins fails therefore to adequately explain target specificity, and so analyses of complexes of NCS proteins with peptides derived from their target proteins have been undertaken in an effort to better address this question. These studies have highlighted both common and distinct aspects of target recognition that help explain both NCS protein promiscuity and specificity. A common feature is that on Ca^2 +^ binding, the NCS proteins undergo a conformational change that exposes a hydrophobic groove within which one or two helices from target proteins can bind. Once Ca^2 +^ loaded the NCS, proteins undergo relatively limited structural changes on target ligand binding unlike the more dramatic changes seen in calmodulin conformation on ligand binding (described below).

**Fig. 3:**
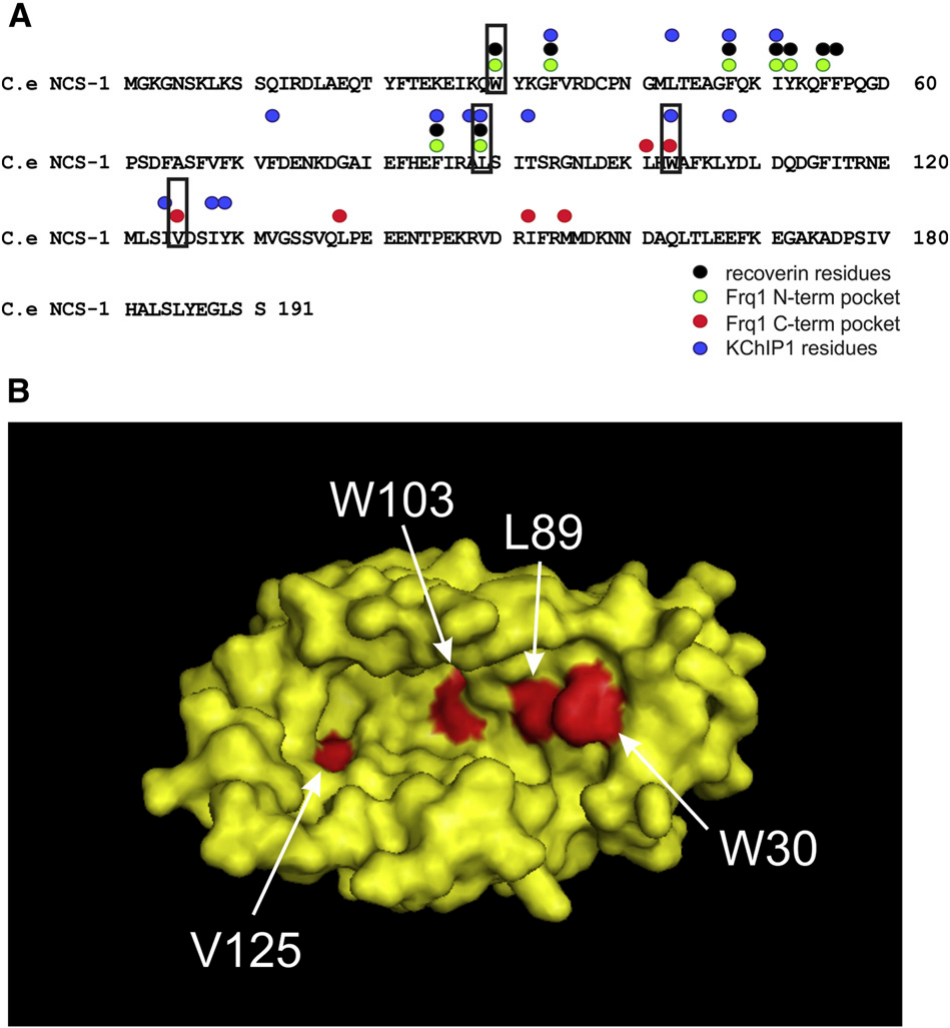
Key residues for target protein interactions in the hydrophobic groove of NCS proteins identified from structural studies and amino acids identified for mutagenesis in *Caenorhabditis elegans* NCS-1. (A) Hydrophobic residues implicated in target protein interactions are conserved in *C. elegans* NCS-1. Those residues that directly make contact with target proteins in structurally characterised complexes are indicated above the sequence of *C. elegans* NCS-1. Residues selected for mutagenesis are boxed. (B) Position of residues selected for mutagenesis in the predicted NCS-1 structure. The selected amino acids are within the hydrophobic groove are shown in red in a surface representation of a model structure for *C. elegans* NCS-1 based on the crystal structure of human NCS-1 (PDB1G8I). Adapted from Martin et al 2013 [60].

Key hydrophobic amino acids within the hydrophobic groove of the NCS proteins that are conserved across the whole NCS family are directly involved in the target interactions in multiple NCS proteins (Fig. 3). Despite this similarity in the mechanism for target protein binding, there are structural differences that will determine specificity [37], [30]. There are differences in the overall size of the exposed hydrophobic groove (which may require some movement of the C-terminal tail of the protein) so that two or only one helix can bind. Also, differences in the distribution of surrounding surface exposed charged residues influences binding of specific ligands. In addition, there are key differences in the role of the C-terminal tail. In the case of KChIP1 [92], [93] and recoverin [94], the C-terminal proximal pocket of the groove is partially occluded by the C-terminal tail of the NCS protein allowing only a single helix to bind. In recoverin, amino acids in the C-terminal tail are actually required to make direct contact with the rhodopsin kinase target peptide for high-affinity binding [95]. In one of the published structures of NCS-1, the C-terminal tail appears to be able to sit in the hydrophobic groove in the absence of ligand [96]. This is likely an auto-regulatory mechanism to control target binding as the groove is fully exposed in another published structure of human NCS-1 [97]. In addition, in the structure of the yeast protein in complex with Pik1, two helices of Pik1 were bound within a fully exposed hydrophobic groove and the C-terminus appeared disordered, suggesting that the C-terminal tail can undergo a conformational change to allow its movement out of the groove on ligand binding [91], [98].

The structural studies on NCS protein/target interaction have been based on the use of short peptides derived from the target protein. These studies have provided a consistent picture in which conserved hydrophobic residues are crucial. Nevertheless, it is important to assess the significance of the structural data using a physiological model. This would, for example, allow a test of requirement for the key residues in the hydrophobic groove and also the requirement for the C-terminal tail for NCS-1 function. This was approached using an assay for temperature-dependent locomotion in *C. elegans* [99] that requires NCS-1 function in the pair of AIY neurons in the worm. Using an NCS-1 null mutant worm strain, an assay was developed for functional reconstitution. Key amino acids were then mutated in the N- and C-terminal parts of the hydrophobic groove (Fig. 3), and the mutant proteins expressed in the null background. The findings from this assay were fully consistent with the structural data and showed that for normal NCS-1 function in this behavioural assay hydrophobic resides in both the N- and C-terminal parts of the hydrophobic groove were required [60]. In addition, full functional reconstitution occurred after truncation of the C-terminus of NCS-1 and indeed truncation of residues 169–191 to remove the entire C-terminal helix resulted in a gain of function [60]. These findings are consistent with the notion that the C-terminal tail of NCS-1 unlike that in for recoverin is not essential for direct target interactions but instead may have an auto-inhibitory role by binding with the C-terminal part of the hydrophobic groove in the absence of a suitable binding ligand [96], and this may prevent binding of inappropriate ligands.

Evidence for a role in specificity determination of residues surrounding the hydrophobic groove has come from analysis of *Drosophila* frequenins. In *Drosophila* species, a gene duplication event generated two genes encoding closely similar proteins known as Frq1 (the originally identified frequenin [63]) and Frq2 [100]. Both isoforms affect synaptic transmission and synaptic bouton number [101]. Frq1 and Frq2 differ by only 10 amino acids, and it was unclear whether they would have distinct functions. Recent work has now identified the guanine nucleotide exchange factor Ric8a as a binding partner for Frq2 but not Frq1 and functional analyses suggest that the interaction is involved in the physiological roles of Frq2 in the fly [102]. The interaction with ric8 was conserved in the human proteins [102]. From examination of the crystal structure of Frq2, a small number of solvent exposed amino acids that differed between the two frequenins were highlighted as being potentially important for target recognition, and mutagenesis of Frq1 established that the amino acids R94 and T138 located at the edge of the hydrophobic groove were required for Ric8a binding. Incidentally, the removal of the C-terminal helix of Frq2 increased its binding to Ric8a consistent with the findings on the functional effect of deletion of the C-terminal tail of NCS-1 in *C. elegans* [60]. These findings provide clear evidence in support of the structural models for NCS protein/target specificity [37].

## 3 Case Study 2—CaBPs, Ca^2 +^ sensors with targets in common with and distinct from calmodulin

**Fig. 4:**
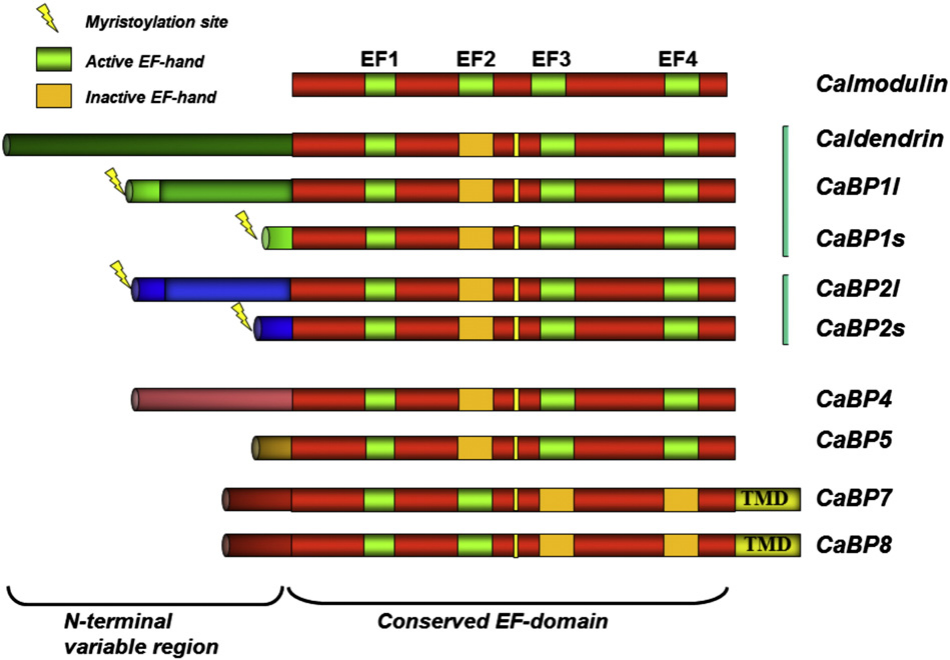
Domain structure of the CaBP family of Ca^2 +^ sensors compared to calmodulin. Unlike calmodulin, each of the CaBPs has at least one inactive EF hand. Some are membrane targeted due to N-terminal myristoylation whereas CaBP7 and CaBP8 possess C-terminal transmembrane domains that determine their membrane association [114], [115].

The CaBP proteins in humans are encoded by 6 functional genes (Fig. 4), with CaBP1 and CaBP2 existing as variants derived from alternative splicing [26], [27], [103], [104]. They have a higher level of similarity to calmodulin than do the NCS proteins but have divergent N-terminal domains that differ between the CaBPs and which do not occur in calmodulin and also have a specific larger linker region between the N- and C-terminal EF hand domains. Certain of the CaBPs can be membrane-associated through N-terminal myristoylation or possession of a C-terminal transmembrane domain (Fig. 4). Both CaBP1/caldendrin and calmodulin can interact with VGCCs. In addition, CaBP1 has been shown to bind and regulate InsP_3_ receptors [105], which also bind calmodulin [106]. Originally, it was suggested that CaBP1 activates the receptor, but subsequent work has established that instead it has an inhibitory effect on receptor function in response to InsP_3_ [107], [108].

CaBP1 is similar to calmodulin in that it has distinct N-and C-terminal lobes [109], [110], [111]. Each contains two EF hands, but unlike calmodulin, the second EF hand is non-functional in CaBP1. The structure of CaBP1 was derived from analyses of the crystal structure of a form with Ca^2 +^-bound to EF3 and EF4 [109], [110], [111] and an NMR solution structure of a form with Ca^2 +^ bound to EF hands 1, 3 and 4 [109], [110], [111]. Unlike in the solution structure, CaBP1 in the crystal structure was found to be oligomerised, and there were some differences in the N-lobe between the two structures. The C-terminal but not the N-terminal lobe binds to the InsP_3_ receptor [110], and the binding interaction and inhibition of receptor function have been shown to require hydrophobic residues that are exposed in the Ca^2 +^-bound form of the C-lobe and that permit binding to complementary hydrophobic regions on the receptor [112]. In addition to characterising the binding interaction, this structural study also suggested a mechanism whereby CaBP1 inhibited InsP_3_ receptor gating by limiting inter-subunit movements to stabilise a closed state of the channel.

**Fig. 5:**
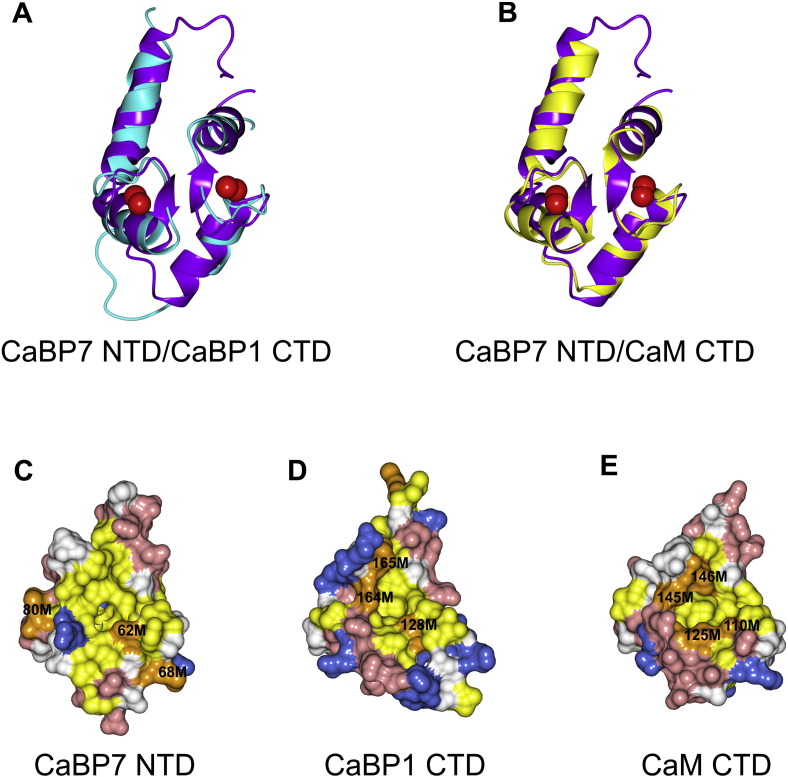
Comparison of the N-terminal domain of CaBP7 with the C-terminal domains of CaBP1 and calmodulin. Ribbon representation of Ca^2 +^-bound CaBP7 NTD (PDB code: 2LV7, residues 30–100, purple) superposed with (A) Ca^2 +^-bound CaBP1 CTD (cyan, PDB code: 2LAP) and (B) Ca^2 +^-bound CaM CTD (yellow, PDB code 1CLL, residues 80–147). Red spheres represent bound Ca^2 +^. Space-filling representations of (C) Ca^2 +^-bound CaBP7 N-terminal domain (PDB code: 2LV7, residues 30–100), (D) Ca^2 +^-bound CaBP1 C-terminal domain (PDB code: 2LAP) and (E) Ca^2 +^-bound CaM C-terminal domain (PDB code: 1CLL, residues 80–147), showing the predicted ligand binding face. Acidic residues and basic residues are shown in red and blue, respectively. Hydrophobic residues are shown in yellow with the exception of Met residues which are highlighted in orange. Adapted from McCue et al 2012 [119].

CaBP7 and CaBP8 (also known as calneurons II and I, respectively [104], [113]) are evolutionarily distinct from the other CaBPs [103] and have a unique C-terminal transmembrane domain [114], [115], [116]. Presently, they have only one known target, phosphatidylinositol-4-kinase IIIβ (PI4KIIIβ), which is also regulated by NCS-1 but is not known to be regulated by other Ca^2 +^ sensors. Whereas NCS-1 stimulates the kinase at elevated [Ca^2 +^]_i_ [117] CaBP7 and CaBP8 inhibit its activity at resting [Ca^2 +^]_I_, and this action can fully explain the physiological effects of CaBP7 and CaBP8 on membrane traffic [118]. The active EF hands in CaBP7 and CaBP8 are present in the N-terminal domain (Fig. 4), and this domain from CaBP7 can independently bind PI4KIIIβ [119]. The overall tertiary structure of the Ca^2 +^-bound N-terminal domain (residues 1–100) of CaBP7 was solved by solution NMR [119]. The structure is most similar to that of the C-terminal lobes of calmodulin and CaBP1 (Fig. 5). Clues to the basis for specificity of target binding come from examination of space-filling representations of the three structures. CaBP7 has an exposed hydrophobic face that is larger in area than that found in calmodulin or CaBP1 with the presence of fewer charged residues [119]. Notably, CaBP7 lacks three of the four methionine residues in the hydrophobic pocket that are present in both the calmodulin N- and C-terminal lobes and that are required for target-binding by calmodulin [24], [120], [121]. Three of these methionines are conserved in the CaBP1 C-terminal lobe, and interestingly, two of these (M164 and M165) make contact with the N-terminal domain of the InsP_3_ receptor in the docked complex [112]. It may be significant that the methionines in CaBP7 and also CaBP8 have been exchanged for conformationally immobile leucine and isoleucine residues, which might help explain the restricted number of targets available for interaction with CaBP7. The underlying hypothesis is that the CaBP hydrophobic binding surface can be made more flexible by increasing the number of methionines present or more rigid by reducing their number and that the degree of flexibility governs target promiscuity. Consistent with this idea, calmodulin has many more known interaction partners (> 300 [24]) than CaBP1 which in turn exhibits a considerably larger interactome (at least 14 targets) than CaBP7, which is currently known to interact only with PI4KIIIβ. It should be noted, however, that we cannot rule out that further work will identify additional targets for CaBP7 and CaBP8.

## 4 Case study 3—regulation of voltage-gated Ca^2 +^ channels by multiple Ca^2 +^ sensors

**Fig. 6:**
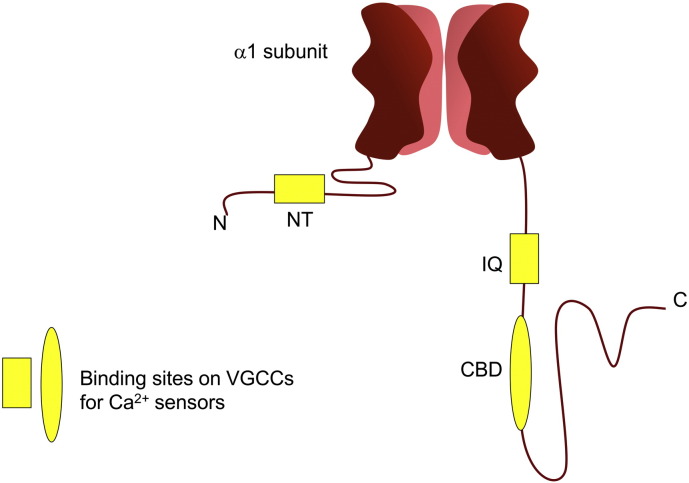
Potential binding sites on VGCCs for Ca^2 +^ sensors that have been identified in the N- or C-terminal cytoplasmic domains of the α_1_ subunits of various types of VGCCs. These include a site in the N-terminus and the IQ and calmodulin-binding (CBD) domains in the C-terminus. Other regions of the C-terminal domain may also be required for Ca^2 +^ sensor interactions.

Ca^2 +^-entry through voltage-gated Ca^2 +^ channels in neurons triggers neurotransmitter release, changes in gene expression, underpins synaptic plasticity and affects neuronal development [122], [123], [124]. The multiple types of VGCCs have been well characterised and found to undergo extensive regulation including feedback by Ca^2 +^ itself leading to rapid Ca^2 +^-dependent facilitation (CDF) or Ca^2 +^-dependent inhibition (CDI) on a millisecond timescale. The speed of this regulation is explained by the response of the channels being due to nanodomains of Ca^2 +^ near the mouth of the channel [125]. Both CDF and CDI are believed to contribute to synaptic plasticity [126], [127], [128]. A recent review has summarised the history of the discovery of VGCC regulation by Ca^2 +^ and progress on the characterisation of the mechanisms particularly those involving calmodulin [129]. The regulation of VGCCs by other Ca^2 +^ sensors has also been the subject of a recent review [128]. Pioneering studies identified calmodulin as a Ca^2 +^ sensor required for CDI of L-type (Ca_V_1.2) Ca^2 +^ channels [130], [131]. It was subsequently demonstrated that Ca^2 +^-free (apo) calmodulin was pre-bound to the pore-forming α_1_ channel subunit and that this was mediated through the so-called IQ domain (Fig. 6) of the α_1_ subunit [132], [133], [134]. The fact that calmodulin is already bound to the channel subunit explains how the channels can be regulated so rapidly in response to Ca^2 +^ elevation in local nanodomains.

**Fig. 7:**
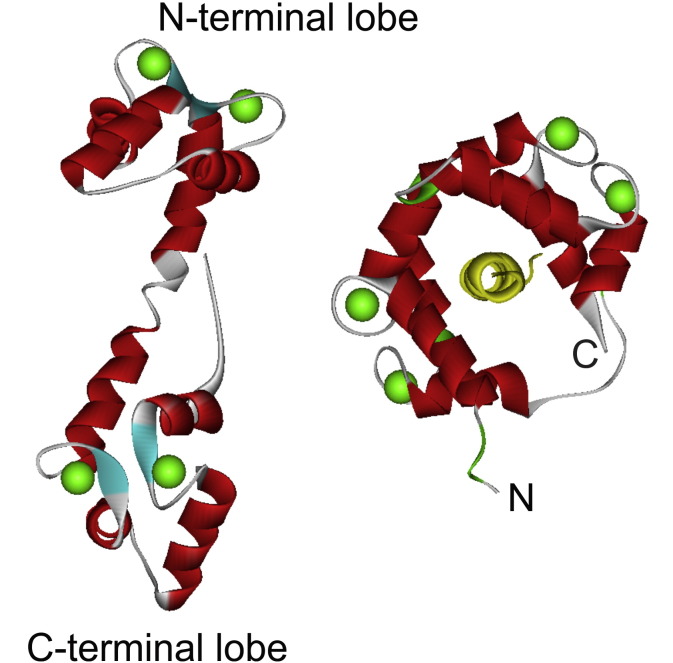
Comparison of the structures of Ca^2 +^-loaded calmodulin with and without bound target peptide. The structures shown are of Ca^2 +^-bound calmodulin alone ([180]) on the left or in a complex with the IQ-like domain of the Ca_v_1.2 Ca^2 +^-channel α_1_-subunit (PDB 2F3Z) [170] on the right with the IQ-like peptide shown in yellow.

A role for calmodulin was subsequently extended to other VGCC types. The use of Ca^2 +^-insensitive calmodulin mutants with inactivated EF hands demonstrated its requirement. Calmodulin has independent N- and C-terminal lobes, which each possess 2 active EF hands. The two lobes can show major structural movement relative to each other on ligand binding allowing them to both bind to the same helix (Fig. 7). For Ca_V_1.2 channels, CDI was mediated through the C-lobe [130]. In contrast, for Ca_V_2.1 [135], [136], Ca_V_2.2 [137] and Ca_V_2.3 [137], it is the N-lobe that controls CDI. Of particular interest is the finding that for P/Q-type (Ca_V_2.1) channels calmodulin is required for both CDI and CDF with the N-lobe of calmodulin involved in CDI and the C-lobe in CDF [135], [136]. The majority of studies have focused on the IQ domain in the C-terminal cytoplasmic region of the subunit (Fig. 6) as the binding site for calmodulin on the various channel types [138], [139]. Other studies have implicated an additional calmodulin-binding domain (CBD) in the C-terminus present in Ca_V_2.1 [136], and there are likely to be additional binding sites in the N-terminal domain of the α_1_ subunit (Fig. 6).

The picture of Ca^2 +^-dependent regulation of VGCCs became more complex when it was discovered that various other Ca^2 +^ sensors could exert differing effects on CDI, CDF (Table 2) or channel current, in some cases apparently also through binding to the IQ domain [128]. The first such indication of an additional regulatory mechanism came from the finding that CaBP1 could elicit CDI itself and also reduce CDF due to calmodulin in Ca_V_2.1 channels [140]. CaBP1, as described above, has been shown to have a bi-lobe structure like calmodulin [109], and it has been suggested that it exerts its effect on CDF by competing-off calmodulin from the IQ domain [109], [141], [142].

Ca_V_2.1 channels appear to be under extensive regulatory control by multiple Ca^2 +^ sensors. In addition to calmodulin and CaBP1, these channels are also regulated by the NCS proteins VILIP-2 and NCS-1. VILIP-2 inhibits the inactivation brought about by calmodulin and also enhances facilitation in the same way as calmodulin [143], [144] through interaction with the IQ and the CBD domains. Electrophysiological experiments implicated NCS-1 in CDF of Ca_V_2.1 channels [145] and also in an autocrine pathway that negatively regulates these P/Q-type Ca^2 +^ channels in adrenal chromaffin cells [146], [147]. In addition, functional effects of loss of NCS-1 (Frq) in Drosophila have been investigated using genetic approaches and attributed to the regulation of cacophony [56], [148], which encodes an α1-subunit of a fly VGCC. Despite these studies, a direct interaction of NCS-1 with the Ca_V_2.1 channel or any other VGCC had not been reported. Recently, however, the use of multiple biochemical and structural approaches has consistently demonstrated a direct interaction of NCS-1 with the Ca_V_2.1 channel α1-subunit that requires the IQ domain but does not appear to involve the CBD in the C-terminus of the α1-subunit [149]. NMR analysis also demonstrated direct binding of an IQ domain peptide to NCS-1.

Members of the CaBP family have been found to regulate a range of VGCC types (Table 2). For example, CaBP1 prolongs Ca_V_1.2 currents and inhibits CDI [150], [151]. The gene encoding CaBP1 also gives rise to a longer isoform, caldendrin, which may be the predominant form in brain [152], [153]. Caldendrin also reduces CDI of Ca_V_1.2 channels but to a lesser extent than CaBP1 [150]. Studies of CaBP5 function in the retina have suggested that it acts as an inhibitor of CDI of Ca_V_1.2 channels [154]. Potentially other cell-specific forms of regulation by multiple CaBPs of Ca_V_1.3 channels have also been discovered in auditory hair cells [155], [156], [157], although CaBP1 may be the principal regulator [128]. In photoreceptors, there is a potential function for CaBP4 in the regulation of Ca_V_1.3 channels [158]. CaBP4 may also be important in photoreceptors for the regulation of Ca_V_1.4 channels through an effect on the voltage activation of these channels [159], [160].

Many of the studies on the regulation of CDF or CDI of VGCCs by Ca^2 +^ sensors have been based on expression of the sensors and VGCC subunits in heterologous cell systems. This, therefore, raises questions about the physiological relevance of the interactions of the Ca^2 +^ sensors for neuronal function. Support for a significant role has come from a study in which expression of modified Ca_V_1.2 channels in superior cervical ganglion (SCG) neurons was used to probe the general role for interaction of Ca^2 +^ sensors in synaptic plasticity. In this study, it was shown that mutation of the IQ (IM in Ca_v_2.1) motif or the CBD that would prevent CDF or CDI reduced facilitation or short-term synaptic depression, respectively [161]. In addition, another study using SCG neurons showed that expression of CaBP1 or VILIP-2 had effects on synaptic plasticity [127] consistent with their effects on CDF and CDI in heterologous models [162].

One problem with the originally proposed competitive model of action for CaBPs in which they need to compete endogenous calmodulin from the IQ domain is that calmodulin is expressed in brain at considerably higher levels than CaBPs. CaBP1 has been shown, however, to interact with an additional site in the N-terminus of Ca_V_1.2 [163]. More recently, it has been suggested that apo-CaBP4 and apo-calmodulin may both be associated with Ca_V_1.3 channels as a consequence of CaBP4 binding to regions other than the IQ domain [156] so that the higher concentration of calmodulin would not be a significant barrier to a physiological role for CaBPs. In addition to these considerations, it should also be emphasised that calmodulin interacts with a large number of cellular targets. Its availability for any given interaction is therefore difficult to accurately determine and this also likely impacts on the ability of other calcium sensors to modulate shared targets.

Findings consistent with an important role for CaBP4 in the regulation of phototransduction have come from the study of a CaBP4 mouse knock-out and also the presence of mutations in human CaBP4 [164], [165] and in Ca_V_1.4 [166], which cause retinal disorders including congenital night blindness. Data from the CaBP5 knock-out mouse support a role for CaBP5 in altering retinal sensitivity though its effects on Ca_V_1.2 channels [154]. In addition, a mutation in CaBP2 resulting in its truncation has been found that results in autosomal hearing impairment. CaBP2 is expressed in cochlear hair cells where the effect of the truncation may be due to impaired suppression of CDI of Ca_v_1.3 channels [167].

The structural basis for how calmodulin or other Ca^2 +^ sensors exert their Ca^2 +^-dependent effects on VGCCs has been a subject of intensive study. Key issues to be resolved include (1) how can the two lobes of calmodulin have divergent functions when both bind to the IQ domain, (2) why does the C-lobe dominate in CDI in Ca_V_1.2 channels but the N-lobe does so in Ca_V_2 channels, (3) how can binding of calmodulin to an intracellular region of the channel α_1_ subunit affect the gating properties of the channel pore and (4) how can additional Ca^2 +^ sensors that mediate differing effects do so particularly in the presence of calmodulin tightly bound to the IQ domain [129], [168]. A major focus for the calmodulin control of VGCCs has been in analysis of the IQ domain interaction. Initial studies characterised the structure of the complex of Ca^2 +^-bound calmodulin with an IQ-like peptide from Ca_V_1.2 (Fig. 7). These indicated that calmodulin wrapped around the IQ helix with both the N- and C-terminal lobes binding and with the helix bound in an unusual parallel manner with the N- and C-lobes of calmodulin binding to the N- and C-terminal ends of the IQ helix [169], [170]. It was subsequently found that calmodulin bound the IQ peptides of Ca_V_2.1, Ca_V_2.2 or Ca_V_2.3 channels in an anti-parallel manner giving a possible explanation for the reverse role of the N- and C-lobes in CDI of the two classes of channels [138]. This explanation was not supported by another study, however, that showed that all IQ/calmodulin interactions had a parallel conformation, although this study was based on complexes with a shorter IQ peptide [139]. More recent studies have begun to explore the structural basis for calmodulin interaction with larger constructs from the C-terminal domain of the VGCCs (reviewed in [168]). It is clear that much still remains to be learnt but recent work has suggested that interactions in the C-terminus outside the IQ domain and with the N-terminus of the α_1_ subunit involving the Ca^2 +^-loaded N-lobe may be important [171].

A full structural characterisation of the interaction of CaBP1 with VGCCs is not available but one analysis has used chimeras with calmodulin and specific mutants to dissect the requirements for the regulation of Ca_V_1.2 channels. This has demonstrated that both lobes of CaBP1 are functionally important with the C-lobe anchoring to the channel subunit as does the C-lobe of calmodulin. The N-lobe and also the inter-lobe linker specific for the CaBPs are required for modification of channel function and this could contribute to the different effects on the channel of CaBP1 compared to calmodulin [109]. It has been suggested that differential effects of calmodulin and CaBP1 may in part be due to the structurally different interactions with the IQ domain [109]. It is also possible that interactions with other regions of the α_1_ subunit may be important, but this will require further structural characterisation. There is currently no published data on the structural bases for the interactions of other Ca^2 +^ sensors with VGCCs.

## 5 Conclusions

One aspect that emerges from consideration of the three case studies above is the variation in level of specificity versus promiscuity of the Ca^2 +^ sensors which is determined by fundamental structural characteristics of the proteins. Calmodulin is the most promiscuous with many target proteins. It consists of two independent lobes joined by a flexible linker that allows calmodulin to wrap around and interact with target proteins in multiple different ways using mutually induced fit to ensure high affinity interaction [172]. Also, calmodulin possesses several residues with flexible side chains on its hydrophobic binding surface that also seems to be important to its promiscuity accommodating binding of numerous different ligands to the same protein surface. The CaBP proteins while superficially similar to calmodulin in having two N- and C-terminal lobes have a reduced range of targets as a consequence of having one or more inactivated EF hands and a reduction to varying levels (see CaBP1 versus CaBP7/8) in the number of methionines presented on their binding surface. They also have specialisations (such as myristoylation or the transmembrane domains of CaBP7 and CaBP8) that will limit their access to potential cytoplasmic target proteins.

On Ca^2 +^ binding, Ca^2 +^ sensors undergo a conformational change that allows them to bind ligands and may then undergo further conformational changes. This in turn will allow them to regulate the function of the target proteins. Most of the available structural characterisation has provided information on the binding mechanism and specificity but so far has provided little information on how the activity of the target protein is regulated. This is particularly notable in the case of VGCCs where even different lobes of calmodulin can have different functional effects after binding to a site on the channel α_1_ subunit some distance from the channel pore. For VGCC regulation, we need further information on the structural basis of interactions of Ca^2 +^ sensors outside of the IQ domain, with the rest of the C-terminus, with sites in the N-terminus of the α_1_ subunit and also potentially with other subunits of the holo-channel. The NCS proteins such as NCS-1 have a smaller range of targets and differ from calmodulin in having two lobes that have a very rigid inter-relationship severely reducing the conformational flexibility between the two lobes. Target binding is due to the exposure of a hydrophobic groove the size and surrounding residues of which in conjunction with a variable and more flexible C-terminal tail determine which ligands can bind. A completely un-investigated aspect of NCS-target interaction is how NCS protein binding influences target conformation and structure and how this translates to function. Again, structural insights in this regard are limited by the complexes characterised to date containing only short peptides derived from the target protein. Further progress in this field will, therefore, require characterisation of Ca^2 +^ sensors complexes with larger, ideally full-length, target proteins in order to contribute to an understanding of the mechanistic basis of differential signal processing in response to Ca^2 +^ signals.

## Figures and Tables

**Table 1:** Key functionally characterised target proteins regulated by NCS-1.

Target	Ca^2 +^-bound or apo	Effect on target	Functional consequences	References
ARF1	Ca^2 +^-bound	Competes for PI4KIIIβ activation	Regulation of TGN to plasma membrane traffic	[117], [173]
α_1_ subunit of Ca_V_2.1	Ca^2 +^-bound	Activates Ca^2 +^-dependent facilitation of channel	Increases facilitation of neurotransmitter release	[145], [174]
Dopamine D2 receptor	Ca^2 +^-bound	Inhibits internalisation of receptor	Promotes spatial memory formation	[70], [74]
ILIRAPL1	Ca^2 +^-bound	?	Regulates N-type channels, secretion and neurite elongation	[84], [175]
PI4KIIIβ	Ca^2 +^-bound	Activates the enzyme	Regulation of TGN to plasma membrane traffic	[64], [173]
Adenosine A2 receptor	Apo	?	Increases receptor signalling	[176]
GRK2	Apo	Inhibits kinase activity	Inhibits receptor internalisation	[74]
InsP_3_ receptor	Apo	Enhances receptor activity	Increases calcium signalling in neurons and heart	[177], [178]
Ric8A	Apo	?	Increases synapse number and synaptic release probability	[102]
TRPC5	Apo	Activates channel	Retards neurite growth	[179]

**Table 2:** Summary of the direct regulation of Ca^2 +^-dependent facilitation (CDF) or Ca^2 +^-dependent inhibition (CDI) of neuronal VGCCs by Ca^2 +^ sensors.

VGCC	Type	CDF	CDI	References
Ca_V_1.2	L-type	CaBP1	CaM, CaBP1↓ Caldendrin↓ CaBP5↓	[130], [131], [150], [151], [154]
Ca_V_1.3	L-type		CaM, CaBP1,2,3,& 4↓	[155], [156], [157], [158], [167]
Ca_V_1.4	L-type		CaBP4↓	[160]
Ca_V_2.1	P/Q-type	CaM, VILIP-2, NCS-1, CaBP1↓	CaM, CaBP1, VILIP-2↓	[135], [136], [140], [143], [145], [146]
Ca_V_2.2	N-type		CaM	[137]
Ca_V_2.3	R-type		CaM	[137]
